# Molecular Characterization Analysis of Thalassemia and Hemoglobinopathy in Quanzhou, Southeast China: A Large-Scale Retrospective Study

**DOI:** 10.3389/fgene.2021.727233

**Published:** 2021-09-30

**Authors:** Jianlong Zhuang, Na Zhang, Yuanbai Wang, Hegan Zhang, Yu Zheng, Yuying Jiang, Yingjun Xie, Dongmei Chen

**Affiliations:** ^1^Prenatal Diagnosis Center, Quanzhou Women’s and Children’s Hospital, Quanzhou, China; ^2^Department of Gynecology, Quanzhou Women’s and Children’s Hospital, Quanzhou, China; ^3^Research and Development Department, Yaneng BIOscience (Shenzhen) Co. Ltd., Shenzhen, China; ^4^Key Laboratory for Major Obstetric Diseases of Guangdong Province, Department of Obstetrics and Gynecology, The Third Affiliated Hospital of Guangzhou Medical University, Guangzhou, China; ^5^Key Laboratory of Reproduction and Genetics of Guangdong Higher Education Institutes, The Third Affiliated Hospital of Guangzhou Medical University, Guangzhou, China; ^6^Department of Neonatal Intensive Care Unit, Quanzhou Women’s and Children’s Hospital, Quanzhou, China

**Keywords:** thalassemia, hemoglobinopathy, molecular spectrum, DNA sequencing, Southeast China

## Abstract

**Background:** There are limited reports available on investigations into the molecular spectrum of thalassemia and hemoglobinopathy in Fujian province, Southeast China. Here, we aim to reveal the spectrum of the thalassemia mutation and hemoglobinopathy in Quanzhou prefecture, Fujian province.

**Methods:** We collected data from a total of 17,407 subjects with the thalassemia trait in Quanzhou prefecture. Gap-PCR, DNA reverse dot blot hybridization, and DNA sequencing were utilized for common and rare thalassemia gene testing.

**Results:** In our study, we identified 7,085 subjects who were carrying thalassemia mutations, representing a detection rate of 40.70% (7,085/17,407). Among them, 13 different α-thalassemia gene mutations were detected, with the most common mutation being –^*SEA*^ (69.01%), followed by –α^3.7^ (21.34%) and –α^4.2^ (3.96%). We also discovered 26 β-thalassemia gene mutations, with the mutations of IVS-II-654 (C > T) (36.28%) and CD41/42(–TCTT) (29.16%) being the most prevalent. Besides, a variety of rare thalassemia variants were identified. Among them, the –^*FIL*^, β^*Malay*^, β^*IVS–I–*130^, and β^*IVS–II–*672^ mutations were identified in Fujian province for the first time. Additionally, we detected 78 cases of hemoglobinopathies, of which Hb Owari was the first reported case in Fujian province and Hb Miyashiro was the first case identified in the Chinese population.

**Conclusion:** Our study indicates that there is a diverse range of thalassemia mutations, and it also reveals the mutation spectrum of rare thalassemia and hemoglobinopathies in Quanzhou, Fujian province. It provides valuable data for the prevention and control of thalassemia in Southeast China.

## Introduction

Thalassemia is a hereditary blood disorder caused by human globin gene synthesis disorders, of which α- and β-thalassemia are the most common genotypes (Weatherall, 2001). It most commonly occurs in Mediterranean countries, the Middle East, the Indian subcontinent, Southeast Asia, and China (Modell and Darlison, 2008; Weatherall, 2008). Thalassemia is mainly distributed in the southern regions of China, especially Guangdong, Guangxi, and Hainan provinces (Zhang et al., 2010; Li et al., 2014; Yao et al., 2014; Yin et al., 2014; He et al., 2018; Lu et al., 2021).

Fujian province, which is located in Southeast China, also displays a high prevalence of thalassemia (Xu et al., 2013; Huang et al., 2019; Zhuang et al., 2020a). Quanzhou prefecture has the largest population in Fujian and possesses high population mobility, which may have led to greater diversity and complexity of thalassemia gene mutations. Recently, more rare or novel thalassemia mutations have been identified in the Quanzhou region (Zhuang et al., 2019, 2020b). To date, no effective medical treatment for thalassemia intermedia or major has been developed. Fetuses with α-thalassemia major usually die *in utero* or shortly after birth, and this condition also often leads to the mortality of the pregnant mother (Vichinsky, 2013). Fetuses with β-thalassemia major usually develop severe progressive anemia after 3–6 months and rarely survive past 5 years of age if not treated with a regular transfusion program and chelation therapy (Liaska et al., 2016). Therefore, thalassemia genetic detection before marriage or pregnancy, as well as prenatal diagnosis, is the only effective intervention to prevent the births of babies with thalassemia major or intermedia. However, there is very little knowledge on the genotypes of thalassemia, and there is a lack of information on the hemoglobinopathy mutation spectrum in the Quanzhou region.

This retrospective study was performed to analyze the spectrum of the thalassemia gene mutation and characterize the genotypes of rare thalassemia and hemoglobinopathy in Quanzhou prefecture. It aims to provide valuable reference data for the prevention and control of thalassemia in Southeast China.

## Materials and Methods

### Study Subjects

A total of 17,407 subjects who were suspected of being thalassemia carriers were recruited at Quanzhou Women’s and Children’s Hospital between January 2013 and March 2021. The age of these subjects ranged from 1 to 67 years old. We performed thalassemia gene detection on all of the subjects who met the following inclusion criteria: (1) routine hematology examination showed abnormal mean corpuscular volume (MCV) <82 fl/or mean corpuscular hemoglobin (MCH) <27 pg; (2) abnormal hemoglobin electrophoresis; (3) parents or siblings carried the thalassemia gene mutation; (4) at least one of the couple was identified as a thalassemia carrier.

### Hematological Analysis and Serum Ferritin Test

Approximately 4 ml of peripheral blood was collected from each subject and anticoagulated with EDTA-K_2_ for routine blood analysis and hemoglobin electrophoresis analysis. We performed routine blood detection on all of the subjects using an automated cell counter (Sysmex XS-1000i; Sysmex Co., Ltd., Kobe, Japan) and analyzed the hemoglobin components by hemoglobin electrophoresis (Sebia, Evry Cedex, France). Positive hematological screening was defined as an MCV of less than 82 fl and/or an MCH concentration of less than 27 pg and/or hemoglobin A2 (HbA2) levels greater than 3.4% or less than 2.6% or an Hb F value of more than 2.0%. All patients with positive hematological analysis results underwent thalassemia gene testing.

For the serum ferritin test, approximately 3 ml of peripheral blood was collected from the patients. Then, it was centrifuged at 3,500 rpm for 10 min to separate the serum. The serum ferritin test was performed with Siemens Healthcare Diagnostics equipment and kit (Siemens, United States) and using the ADVIA Centaur XP Immunoassay System (Siemens, United States).

### Molecular Diagnosis of Thalassemia

For each subject with positive hematological analysis results for the molecular analysis of common α- and β-thalassemia, we collected a further 2 ml of peripheral blood. An automatic nucleic acid extractor (Ruibao Biological Co., Ltd., Taiwan) was used to extract the genomic DNA of the subjects. We also used Gap-PCR to detect the three common deletional α-thalassemia mutations [Yaneng BIOscience (Shenzhen) Co. Ltd., Shenzhen]. The PCR reverse dot hybridization technique (PCR-RDB) was utilized to detect the three common non-deletional α-thalassemia mutations and 17 common β-thalassemia mutations [Yaneng BIOscience (Shenzhen) Co. Ltd., Shenzhen]. The β-thalassemia mutations we detected were as follows: CD41-42(-TCTT), IVS-II-654(C > T), –28 (A > G), CD71/72(+ A), CD17(AAG > TAG), CD26(GAG > AAG), CD43(GAG > TAG), –29(A > G), CD31(-C), –32(C > A), IVS-I-1(G > T), CD27/28(+ C), –30(T > C), CD14-15(+ G), Cap + 40-43(–AAAC), initiation codon(ATG > AGG), and IVS-I-5(G > C). The experimental operations were performed strictly according to the protocols of the manufacturers.

### Rare Thalassemia Analysis and DNA Sequencing

Rare α-thalassemia genotype screening kits (–^*THAI*^, –α^27.6^, −α^21.9^) and rare β-thalassemia genotype screening kits [Taiwanese, ^*G*^γ^+^(^*A*^γδβ)^0^, SEA-HPFH] were utilized for suspected carriers of rare α- or β-thalassemia deletion. Indicators included low levels of Hb A2 without common α-thalassemia variants, as well as high or low levels of Hb A2 without common β-thalassemia variants.

Gap-PCR was performed to identify the deletion of –^*FIL*^. We designed specific primers according to the known DNA sequences around the breakpoints. These primer sequences were P1: TCTAAAAATTCATCCTTAAGAGAATAA and P2: GATCTAATGTAGTAGAGATAATAACCTTTA. All primers were synthesized at Sangon Biotech (Shanghai). The amplification conditions were 96°C for 5 min, then 35 cycles of 98°C for 30 s, 60°C for 1 min, 72°C for 2 min, and finally 72°C for 10 min. Subsequently, we performed an electrophoresis analysis.

We employed a multiplex ligation-dependent probe amplification (MLPA) assay using the SALSA MLPA Probemix P140-C1HBA (MRC-Holland, Amsterdam, Netherlands) to detect the known or unknown globin gene deletions. DNA sequencing was performed when we observed suspected carriers of rare thalassemia mutations.

### Statistical Analysis

The statistical analysis was conducted using SPSS19.0 software. The measurement data were expressed as $\bar{\text{X}}$ ± s, and we utilized the independent sample *t*-test to compare the means of the two groups. We also applied the chi-square test to compare the detection rates between the groups. A value of *p* < 0.05 was considered statistically significant.

## Results

**FIGURE 1:**
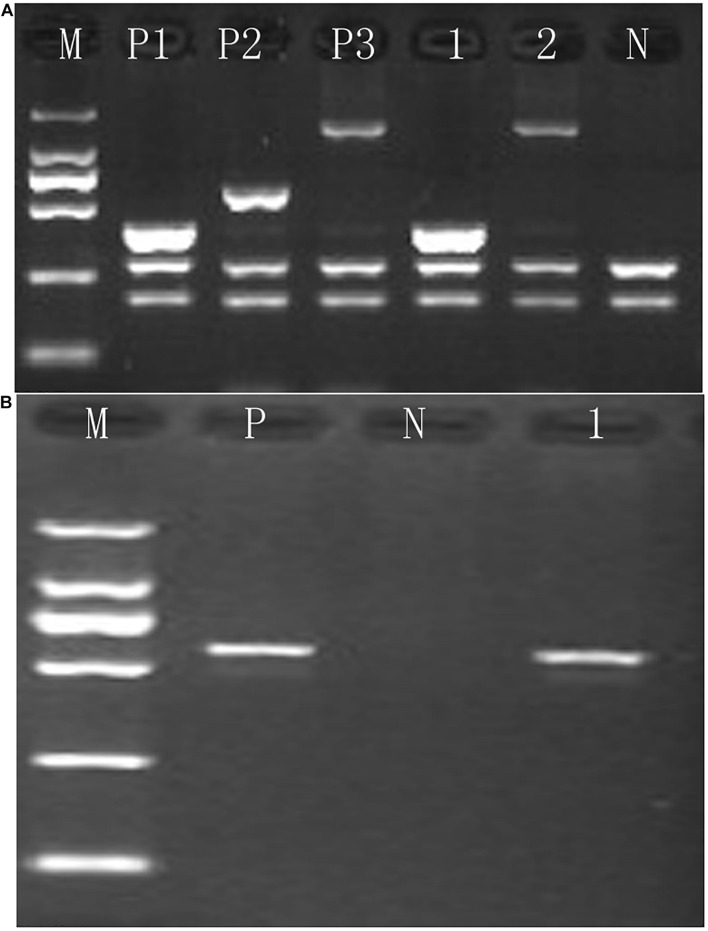
Identification of rare α-thalassemia using gap-PCR. **(A)** Electrophoresis result of –^*THAI*^/αα and –α^27.6^/αα thalassemia; M, maker; P1, positive control of –^*THAI*^/αα; P2, positive control of –α^21.9^/αα; P3, positive control of –α^27.6^/αα; N, negative control; 1, –^*THAI*^ thalassemia carrier; 2, –α^27.6^ thalassemia carrier. **(B)** Electrophoresis result of –^*FIL*^/αα thalassemia; M, maker; P, positive control of –^FIL^/αα; N, negative control; 1, –^FIL^/αα thalassemia carrier.

**FIGURE 2:**
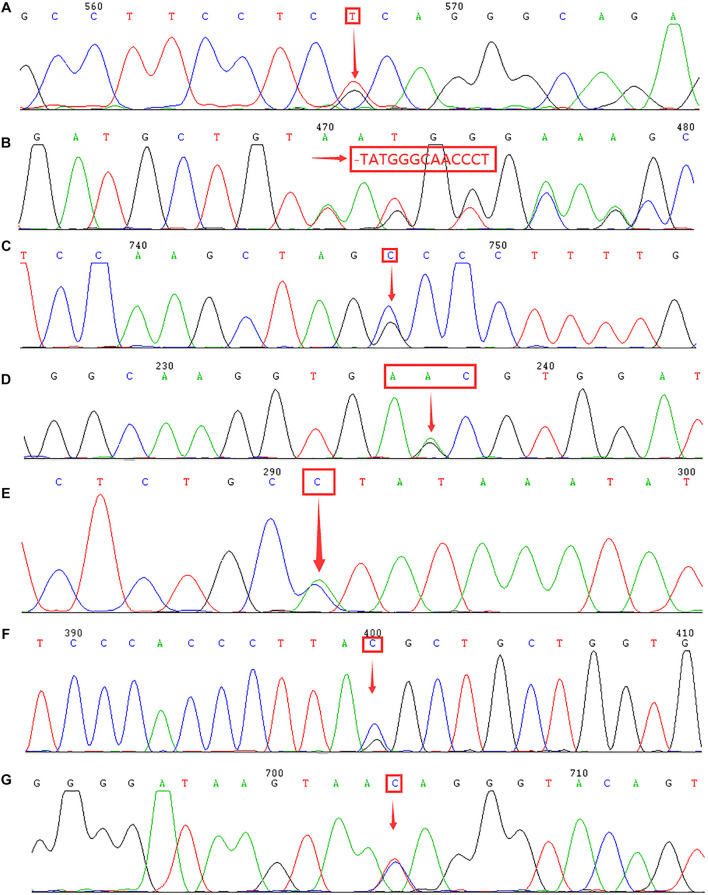
Identification of rare α- and β-thalassemia mutations using DNA sequencing. Arrows indicate the location of the mutations. **(A)** IVS-II-55(T > G) mutation in the *HBA2* gene. **(B)** CD54-58(-TATGGGCAACCCT) mutation in the *HBB* gene. **(C)** IVS-II-806(G > C) mutation in the *HBB* gene. **(D)** Hb Malay(AAC > AGC) mutation in the *HBB* gene. **(E)** IVS-II-672(A > C) mutation in the *HBB* gene. **(F)** IVS-I-130(G > C) mutation in the *HBB* gene. **(G)** IVS-II-81(C > T) mutation in the *HBB* gene.

Of the 17,407 suspected cases, 4,884 subjects were diagnosed with α-thalassemia, including 31 genotypes. Of these, the –^*SEA*^/αα (70.17%) mutation was the most common deletional mutation, followed by –α^3.7^/αα (18.37%) and –α^4.2^/αα (3.34%). The three most common non-deletional mutations were αα^*QS*^/αα, αα^*CS*^/αα, and αα^*WS*^/αα, with frequencies of 2.05%, 1.31%, and 0.94%, respectively. Besides, nine genotypes of rare α-thalassemia were detected, of which –^*THAI*^/αα, Hkαα/–^*SEA*^, and αα^*IVS–II–*55(T > *G)*^/αα were the most common. Of these nine genotypes, our study was the first to detect a –^*FIL*^/αα case in Fujian province, while –α^27.6^/–^*SEA*^ and αα^*IVS–II–*55(T > *G)*^/αα had never before been identified in Quanzhou prefecture (Table 1 and Figures 1, 2).

**TABLE 1:** Distribution of α-thalassemia genotypes in Quanzhou Prefecture.

Genotypes	Cases	Frequency	Class
–^*SEA*^/αα	3,427	70.17%	Common
–α^3.7^/αα	897	18.37%	Common
–α^4.2^/αα	163	3.34%	Common
αα^*QS*^/αα	100	2.05%	Common
–α^3.7^/–^*SEA*^	71	1.45%	Common
αα^*CS*^/αα	64	1.31%	Common
αα^*WS*^/αα	46	0.94%	Common
–α^3.7^/–α^3.7^	30	0.61%	Common
–α^4.2^/–^*SEA*^	16	0.33%	Common
αα^*WS*^/–α^3.7^	11	0.23%	Common
–α^3.7^/–α^4.2^	8	0.16%	Common
αα^*CS*^/–^*SEA*^	7	0.14%	Common
Hkαα/–^*SEA*^	5	0.10%	Rare
–^*THAI*^/αα	5	0.10%	Rare
αα^*IVS–II–*55(T > *G)*^/αα	5	0.10%	Rare
αα^*WS*^/–^*SEA*^	4	0.08%	Common
αα^*QS*^/–^*SEA*^	4	0.08%	Common
αα^*QS*^/–α^3.7^	3	0.06%	Common
αα^*QS*^/–α^4.2^	2	0.04%	Common
αα^*CS*^/–α^3.7^	2	0.04%	Common
–α^4.2^/–α^4.2^	2	0.04%	Common
αα^*WS*^/–α^4.2^	2	0.04%	Common
Hkαα/αα	2	0.04%	Rare
αα^*WS*^/αα^*WS*^	1	0.02%	Common
αα^*CS*^/αα^*WS*^	1	0.02%	Common
αα^*CS*^/–α^4.2^	1	0.02%	Common
–α^3.7^/–^*THAI*^	1	0.02%	Rare
αα/ααα^*anti*4.2^	1	0.02%	Rare
–^*FIL*^/αα	1	0.02%	Rare
–α^27.6^/–^*SEA*^	1	0.02%	Rare
–α^6.9^/–^*SEA*^	1	0.02%	Novel
Total	4,884	100%	

To further analyze the hematological phenotype αα^*IVS–II–*55(T > *G)*^/αα, we performed a comparison of the hematological parameters among the different genotypes of silent α-thalassemia. As Table 2 illustrates, there are significant differences among the silent α-thalassemia groups in the hematological parameters of Hb, MCV, and MCH. Among them, αα^*QS*^/αα-positive cases exhibited lower levels of Hb, MCV, and MCH than those of other groups, and the hematological phenotype was similar to that of α-thalassemia minor. Conversely, the rare genotype αα^*IVS–II–*55(T > *G)*^/αα displayed a milder hematological phenotype similar to αα^*WS*^/αα.

**TABLE 2:** Comparison of hematological parameters among α-thalassemia silent carriers.

Groups	Cases		RBC (× 10^12^/L)		Hb (g/L)		MCV (fl)	MCH (pg)
			
	M	F	M	F	M	F		
αα^*QS*^/αα	23	27	5.51 ± 0.48	4.65 ± 0.39	138.5 ± 10.0	108.5 ± 5.8	75.20 ± 3.44	23.96 ± 1.43
αα^*CS*^/αα	9	21	5.62 ± 0.08	4.44 ± 0.37	147.2 ± 5.6	117.7 ± 7.4	80.77 ± 4.08	26.34 ± 1.02
αα^*WS*^/αα	6	8	5.67 ± 0.16	4.32 ± 0.28	151.3 ± 3.1	126.6 ± 5.2	82.21 ± 4.13	27.57 ± 1.80
αα^*IVS–II–*55(T > *G)*^/αα	2	2	5.23 ± 0.40	4.25 ± 0.12	150.5 ± 6.4	126.0 ± 7.1	84.00 ± 2.71	29.28 ± 0.93
–α^4.2^/αα	17	51	5.65 ± 0.28	4.53 ± 0.35	148.9 ± 6.4	119.3 ± 7.8	80.79 ± 3.81	26.56 ± 1.02
–α^3.7^/αα	42	160	5.63 ± 0.35	4.56 ± 0.36	152.6 ± 7.6	119.9 ± 7.7	80.45 ± 3.06	26.53 ± 1.11
F			0.899	1.722	10.130	13.185	24.014	50.875
P			0.485	0.130	<0.001	<0.001	<0.001	<0.001

*M, male; F, female; Hb, hemoglobin; MCH, mean corpuscular hemoglobin; MCV, mean corpuscular volume; RBC, red blood cell.*

Additionally, 2,056 cases were diagnosed with β-thalassemia. Of these, there were 2,035 cases with β-thalassemia minor and 21 cases with β-thalassemia intermedia or major. The most common β-thalassemia variants were β^*IVS–II–*654^/β^*N*^ (36.19%) and β^*CD*41–42^/β^*N*^ (29.82%), followed by β^*CD*17^/β^*N*^ (16.78%), β^*CD*26^/β^*N*^ (5.35%), and β^–28^/β^*N*^ (4.57%). These five common mutations accounted for 92.71% of β-thalassemia gene mutations. In this study, 17 cases of rare β-thalassemia variants were identified, of which β^*CD*54–58^/β^*N*^ and β^*IVS–II–*806^/β^*N*^ mutations were the first to be reported in Quanzhou, while β^*Malay*^/β^*N*^ and β^*IVS–II–*672^/β^*N*^ had not previously been identified in Fujian province. Moreover, one subject carried two rare β-thalassemia mutations (β^*IVS–I–*130^/β^*IVS–II–*81^), which has never been encountered before (Table 3 and Figure 2).

**TABLE 3:** Distribution of β-thalassemia genotypes in Quanzhou Prefecture.

Types	Genotypes	Cases	Frequency	Class
β^0^/β^*N*^or β^+^/β^*N*^	β^*IVS–II–*654^/β^*N*^	744	36.19%	Common
	β^*CD*41–42^/β^*N*^	613	29.82%	Common
	β^*CD*17^/β^*N*^	345	16.78%	Common
	β^*CD*26^/β^*N*^	110	5.35%	Common
	β^–28^/β^*N*^	94	4.57%	Common
	β^*CD*27/28^/β^*N*^	47	2.29%	Common
	β^*CD*71–72^/β^*N*^	22	1.07%	Common
	β^*CD*43^/β^*N*^	18	0.88%	Common
	β^–29^/β^*N*^	8	0.39%	Common
	β^*CAP+*40–43^β^*N*^	7	0.34%	Common
	β^*Int*^/β^*N*^	4	0.19%	Common
	β^*SEA–HPFH*^/β^*N*^	3	0.15%	Rare
	β^*IVS–I–*1^/β^*N*^	3	0.15%	Common
	β^*Term CD+*32^/β^*N*^	3	0.15%	Rare
	β^*CD*53^/β^*N*^	2	0.10%	Rare
	β^*CD*37^/β^*N*^	2	0.10%	Rare
	β^*IVS–I–*5^/β^*N*^	2	0.10%	Common
	β^*CD*14–15^/β^*N*^	2	0.10%	Common
	β^–90^/β^*N*^	1	0.05%	Rare
	β^*CD*54–58^/β^*N*^	1	0.05%	Rare
	β^*Malay*^/β^*N*^	1	0.05%	Rare
	β^*CD*3^/β^*N*^	1	0.05%	Rare
	β^*IVS–II–*806^/β^*N*^	1	0.05%	Rare
	β^*IVS–II–*672^/β^*N*^	1	0.05%	Rare
β^+^/β^+^or β^0^/β^+^or β^0^/β^0^	β^*IVS–II–*654^/β^*IVS– II –*654^	4	0.19%	Common
	β^*IVS–II–*654^/β^*CD*17^	4	0.19%	Common
	β^*CD*41–42^/β^*CD*41–42^	2	0.10%	Common
	β^*CD*26^/β^*CD*26^	2	0.10%	Common
	β^*CD*17^/β^*CD*17^	1	0.05%	Common
	β^*IVS–II–*654*M*^/β^–28^	1	0.05%	Common
	β^*IVS–II–*654^/β^*CD*27/28^	1	0.05%	Common
	β^*IVS–II–*654^/β^*CD*41–42^	1	0.05%	Common
	β^*CD*41–42^/β^*CD*17^	1	0.05%	Common
	β^*CD*41–42^/β^*CD*26^	1	0.05%	Common
	β^*IVS–II–*654*M*^/β^*CD*26^	1	0.05%	Common
	β^*CD*41–42^/β^*CD*43^	1	0.05%	Common
	β^*IVS–I–*130^/β^*IVS–II–*81^	1	0.05%	Rare
	Total	2056	100%	

Among the suspected cases, 145 were diagnosed with compound α and β-thalassemia, while 34 of these subjects possessed the two most prevalent genotype mutations, –^*SEA*^/αα/β^*IVS–II–*654^/β^*N*^ and –α^3.7^/αα/β^*CD*41–42^/β^*N*^ (Table 4).

**TABLE 4:** Distribution of compound α and β-thalassemia in Quanzhou Prefecture.

Genotypes	–^*SEA*^/	–α^3.7^/	–α^4.2^/	αα^*WS*^/	αα^*CS*^/	αα^*QS*^/	–α^3.7^/–^*SEA*^	–α^3.7^/–α^3.7^
	αα	αα	αα	αα	αα	αα		
β^*IVS–II–*654^/β^*N*^	21	10	3	3	2	2	0	1
β^*CD*41–42^/β^*N*^	13	14	4	4	0	1	0	0
β^*CD*17^/β^*N*^	10	13	1	2	3	2	0	0
β^*CD*26^/β^*N*^	3	5	0	2	0	0	1	0
β^–28^/β^*N*^	3	6	1	0	1	0	0	0
β^*CD*27/28^/β^*N*^	0	2	0	0	0	0	0	0
β^*CD*71–72^/β^*N*^	0	3	0	0	1	0	0	0
β^*CAP+*40–43^β^*N*^	4	0	1	0	0	0	1	0
β^*Int*^/β^*N*^	0	1	0	0	0	0	0	0
β^*CD*26^/β^*CD*26^	0	0	0	0	1	0	0	0

**FIGURE 3:**
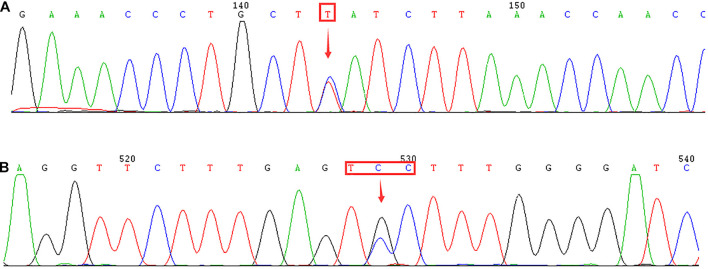
Identification of δ-globin gene mutation using DNA sequencing. Arrows indicate the location of the mutations. **(A)** –77(T > C) (HBD:c.-127T > C) mutation in the *HBD* gene. **(B)** Novel mutation of CD44(TCC > TGC) (HBD:c.134C > G) in the *HBD* gene.

Moreover, 11 subjects with low levels of Hb A2 or β-thalassemia carrier with normal levels of Hb A2 were suspected of having the δ-globin gene mutation. We confirmed that none of these subjects were suffering from iron deficiency anemia. Subsequently, we performed DNA sequencing to detect the HBD gene and discovered two δ-globin gene mutations. Two cases of known mutations [–77(T > C)] and one case of the novel δ-globin gene mutation [CD44(TCC > TGC) (HBD:c.134C > G)] were identified (Figure 3).

In this study, 39 types of mutations were identified in the allele frequencies of α(β) thalassemia mutation chromosomes, including 13 α-thalassemia gene mutations and 26 β-thalassemia mutations. Of the α-thalassemia mutant chromosomes, 5,205 chromosomes carried α-thalassemia gene mutations, of which the most frequent mutation was –^*SEA*^ (69.01%), followed by –α^3.7^ (21.34), –α^4.2^ (3.96%), αα^*QS*^ (2.19%), αα^*CS*^ (1.59%), and αα^*WS*^ (1.48%) (Table 5). Regarding the β-thalassemia mutant chromosomes, 2,223 chromosomes carrying β-thalassemia gene mutations were detected, of which the five most common mutations were IVS-II-654(C > T), CD41-42(-TCTT), CD17(A > T), CD26(G > A), and −28(A > G). The allele frequencies were 36.08%, 29.55%, 17.23%, 5.80%, and 4.77%, respectively (Table 6).

**TABLE 5:** Allele frequency of α-thalassemia mutations in Quanzhou Prefecture.

Mutation type	HGVS name	Allele	Frequency
_*SEA*	NC_000016.9:g.215400_234700del	3592	69.01%
–α^3.7^	NC_000016.9:g.223300_227103del	1111	21.34%
–α^4.2^	NC_000016.9:g.219817_(223755_224074)del	206	3.96%
αα^*QS*^	HBA2: c.377T > C	114	2.19%
αα^*CS*^	HBA2: c.427T > C	83	1.59%
αα^*WS*^	HBA2: c.369C > G	77	1.48%
Hkαα	/	7	0.13%
–^*THAI*^	NC_000016.9:g.199800_233300del	6	0.12%
αα^*IVS–II–*55(T > *G)*^	HBA2: c.300 + 55T > G	5	0.10%
ααα^*anti*4.2^	/	1	0.02%
–^*FIL*^	NC_000016.9:g.200820_232670del	1	0.02%
–α^27.6^	NC_000016.9:g.198215_225854del	1	0.02%
–α^6.9^	NG_000006.1:g.29785_36746del	1	0.02%
Total		5205	100%

*HGVS, human genome variation society.*

**TABLE 6:** Allele frequency of β-thalassemia mutations in Quanzhou Prefecture.

Mutation type	HGVS name	Allele	Frequency
IVS-II-654(C > T)	HBB: c.316–197C > T	802	36.08%
CD41-42(-TCTT)	HBB: c.126_129delCTTT	657	29.55%
CD17(A > T)	HBB: c.52A > T	383	17.23%
CD26(G > A)	HBB: c.79G > A	129	5.80%
–28(A > G)	HBB: c.-78A > G	106	4.77%
CD27/28(+ C)	HBB: c.84_85insC	50	2.25%
CD71-72 (+ A)	HBB: c.216_217insA	26	1.17%
CD43 (G > T)	HBB: c.130G > T	19	0.85%
CAP + 40-43(-AAAC)	HBB: c.-11_-8delAAAC	13	0.58%
–29(A > G)	HBB: c.-79A > G	8	0.36%
Initiation codon(T > G)	HBB: c.2T > G	5	0.22%
SEA-HPFH	NC_000011.10:g.5222877_525	3	0.13%
	0288del		
Term CD + 32(A > C)	HBB: c. + 32A > C	3	0.13%
CD53(-T)	HBB: c.162delT	3	0.13%
CD37(G > A)	HBB: c.113G > A	2	0.09%
IVS-I-1(G > T)	HBB: c.92 + 1G > T	2	0.09%
CD14-15(+ G)	HBB: c.45_46insG	2	0.09%
IVS-I-5(G > C)	HBB: c.92 + 5G > C	2	0.09%
–90(C > T)	HBB: c.-140C > T	1	0.04%
CD3(C > T)	HBB: c.10C > T	1	0.04%
CD54-58(-TATGGG	HBB: c.165_177 delT	1	0.04%
CAACCCT)	ATGGGCAACCCT		
IVS-II-806(G > C)	HBB: c.316–45G > C	1	0.04%
IVS-I-130(G > C)	HBB: c.93-1G > C	1	0.04%
IVS-II-81(C > T)	HBB: c.315 + 81C > T	1	0.04%
Hb Malay(A > G)	HBB: c.59A > G	1	0.04%
IVS-II-672 (A > C)	HBB: c.316–179 A > C	1	0.04%
Total		2,223	100%

*HGVS, human genome variation society.*

**FIGURE 4:**
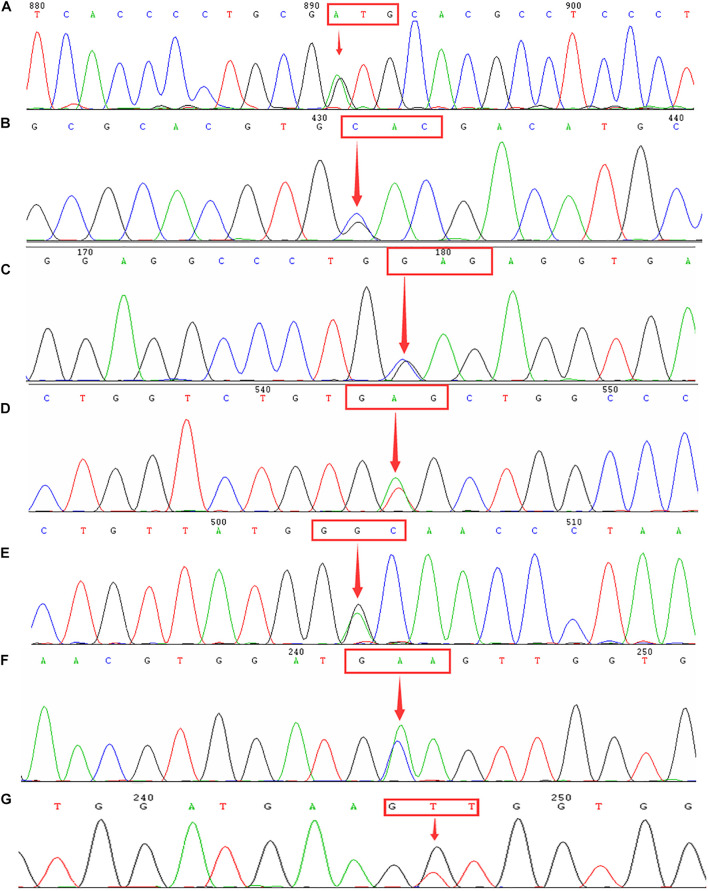
Identification of hemoglobinopathies using DNA sequencing. Arrows indicate the location of the mutations. **(A)** Hb Owari (GTG > ATG at codon 121) mutation in the *HBA1* gene. **(B)** Hb Q-Thailand (GAC > CAC at codon 74) mutation in the *HBA1* gene. **(C)** Hb G-Honolulu (GAG > CAG at codon 30) mutation in the *HBA2* gene. **(D)** Hb New York (GTG > GAG at codon 113) mutation in the *HBB* gene. **(E)** Hb J-Bangkok (GGC > GAC at codon 56) mutation in the *HBB* gene. **(F)** Hb G-Coushatta (GAA > GCA at codon 22) in the *HBB* gene. **(G)** Hb Miyashiro (GTT > GGT at codon 23) mutation in the *HBB* gene.

To further investigate the hemoglobin variants, we performed a DNA sequencing analysis. Altogether, we detected 24 cases of Hb Q-Thailand [CD74(GAC > CAC)], two cases of Hb G-Honolulu [CD30(GAG > CAG)], and one case of Hb Owari [CD121(GTG > ATG)], all of which were induced by the α-globin gene mutation. Similarly, we identified 37 cases of Hb New York [CD113(GTG > GAG)], 12 cases of Hb J-Bangkok [CD56(GGC > GAC)], one case of Hb Miyashiro [CD23(GTT > GGT)], and one case of Hb G-Coushatta [CD22(GAA > GCA)]. These cases were caused by the β-globin gene mutation (Figure 4).

By analyzing the spectrum of thalassemia genotypes in Fujian and the neighboring provinces, we discovered that the highest frequency genotypes of deletional α-thalassemia were similar. However, the mutations in α-thalassemia and β-thalassemia showed distinct regional differences. As Table 7 reveals, αα^*QS*^ was the most common non-deletional α-thalassemia variant in Fujian and Jiangxi province, Guangdong and Hunan province mainly exhibited the αα^*CS*^ mutation, and αα^*WS*^ was the most widespread non-deletional α-thalassemia variant in Guangxi province. The IVS-II-654(C > T) and CD41-42(-TCTT) mutations were the most common β-thalassemia mutations in Fujian and its neighboring provinces, except in Guangxi province, which mainly carried CD41-42(-TCTT) and CD17(A > T) mutations.

**TABLE 7:** Comparison of allele frequency of thalassemia mutations in Fujian and neighboring provinces.

Genotypes	Fujian Province				Guangdong Province (Xu et al., 2004)	Guangxi Province (Zheng et al., 2011)	Jiangxi Province (Lin et al., 2014)	Hunan Province (Liu et al., 2019)
	
	Quanzhou (this study)	Fuzhou (Cao et al., 2019)	Longyan (Dai et al., 2017)	Nanping (Chen M. F. et al., 2020)				

α-thalassemia								
–^*SEA*^	69.01%	77.58%	81.76%	72.68%	48.54%	68.50%	66.51%	52.88%
–α^3.7^	21.34%	14.50%	13.27%	15.30%	36.40%	16.36%	22.86%	31.73%
–α^4.2^	3.96%	3.29%	4.97%	4.10%	11.09%	8.40%	8.08%	9.11%
αα^*QS*^	2.19%	2.69%	–	2.46%	0.42%	0.62%	1.62%	1.15%
αα^*CS*^	1.59%	0.45%	–	0.82%	2.09%	3.03%	0.92%	3.25%
αα^*WS*^	1.48%	1.05%	–	0.55%	–	3.09%	–	1.88%

β-thalassemia								

IVS-II-654(C > T)	36.08%	41.81%	44.95%	35.27%	24.75%	5.26%	40.70%	38.89%
CD41-42(-TCTT)	29.55%	36.10%	28.11%	25.12%	36.36%	48.37%	30.81%	27.49%
CD17(A > T)	17.23%	9.74%	9.08%	10.14%	4.04%	27.40%	5.23%	13.82%
CD26(G > A)	5.80%	1.90%	1.46%	4.35%	5.05%	4.51%	0.58%	3.13%
–28(A > G)	4.77%	4.28%	8.20%	9.18%	16.67%	5.35%	15.70%	4.70%
CD27/28(+ C)	2.25%	4.04%	5.71%	3.38%	2.02%	0.08%	5.23%	2.14%

## Discussion

In China, there is a high prevalence of thalassemia in the regions south of the Yangtze River, particularly in Guangdong and Guangxi. In 2019, a study showed that the prevalence of thalassemia in Fujian was 6.8% (Huang et al., 2019). Few studies are available on the genotypes of thalassemia in Quanzhou prefecture, and there have been few investigations into rare thalassemia and hemoglobinopathy. In this study, we present the spectrum mutation of rare thalassemia and hemoglobinopathy in Quanzhou, Southeast China.

We discovered 7,085 subjects who harbored thalassemia mutations; therefore, the detection rate was 40.70%. As Table 5 displays, we identified 5,205 chromosomes carrying α-thalassemia gene mutations. The three most common deletional variants were –^*SEA*^, –α^3.7^, and –α^4.2^, which is consistent with previous studies in Fujian province and neighboring provinces (Xu et al., 2004; Zheng et al., 2011; Lin et al., 2014, 2019; Dai et al., 2017; Cao et al., 2019; Liu et al., 2019; Chen M. F. et al., 2020). However, the non-deletional variants of α-thalassemia showed a great disparity with other regions (Xu et al., 2004; Zheng et al., 2011; Lin et al., 2014; Liu et al., 2019). Among these variants, 22 cases of rare α-thalassemia were detected, with –^*THAI*^/αα and Hkαα/–^*SEA*^ being the most common. This is consistent with another study conducted in Fujian province (Huang et al., 2019). Before this study, –^*FIL*^/αα had only ever been detected in Taiwan; thus, this was the first-ever reported case in Fujian province (Chen et al., 2002). The –^*FIL*^ deletion covers both the α1 and α2 gene, which leads to α-thalassemia major if compounded with –^*SEA*^ deletion. We identified five cases of the αα^*IVS–II–*55(T > *G)*^/αα mutation in this study, a mutation that was first identified in Fuzhou city and subsequently reported in the Nanping region of Fujian province (Cao et al., 2019; Chen M. F. et al., 2020). This indicates that αα^*IVS–II–*55(T > *G)*^ may be an increasingly common mutation that will possibly become more prevalent in Fujian province. Further analysis of the hematological phenotype of αα^*IVS–II–*55(T > *G)*^/αα provided similar results as tests for αα^*WS*^/αα.

In this study, we detected 2,223 chromosomes carrying β-thalassemia gene mutations. The most frequent mutation was IVS-II-654(C > T), which was consistent with several regions in South China (Lin et al., 2014; Dai et al., 2017; Cao et al., 2019; Liu et al., 2019; Chen M. F. et al., 2020). However, the most prevalent β-thalassemia mutation in the nearby provinces of Guangdong and Guangxi is CD41-42(-TCTT) (Xu et al., 2004; Zheng et al., 2011). Our study indicates a higher frequency of the CD26(G > A) mutation in comparison with other regions in Fujian province. A previous study demonstrated that CD26(G > A) is the most common β-thalassemia mutation in Yunnan province, which is located in Southwestern China (Zhang et al., 2012). In this research, we detected a diverse range of rare or novel β-thalassemia variants. Among them, this was only the second time the β^*IVS–II–*806^/β^*N*^ mutation, which was first identified in the Nanping region north of Fujian, had been identified in humans (Chen M. F. et al., 2020). Additionally, we made the earliest discovery of the rare mutations of Hb Malay(AAC > AGC), IVS-II-672(A > C), and IVS-I-130(G > C) in Fujian province. In the Chinese population, Hb Malay is an extremely rare “Hb Knossos-like” β^+^-thalassemia abnormality, with only one known recorded case (Ma et al., 2000). This mutation is thought to create an alternate splicing site between codons 17 and 18, reducing the efficiency of the normal donor splice site at IVS-I to about 60% (Yang et al., 1989). The IVS-II-672(A > C) mutation had only been previously reported in Guangxi province, which suggests that it is a silent mutation, as the red blood cell indices are normal and there are normal or borderline Hb A2 levels (Zhao et al., 2016). However, in the case of our study, low levels of hemoglobin (106 × 10^12^ g/L) and higher levels of Hb A2 (4.5%) were observed. Thus, more research should be conducted to reveal whether the IVS-II-672(A > C) mutation causes β^+^-thalassemia. Additionally, we identified a rare compound mutation, which is a combination of the IVS-I-130(G > C) (β^0^-thalassemia) and IVS-II-81(C > T) mutations. This causes minor thalassemia, which suggests that the IVS-II-81(C > T) mutation may be a silent mutation.

Besides, we identified two δ-globin gene mutations, including -77(T > C) and CD44 (TCC > TGC) (HBD:c.134C > G). Previous studies have shown that the -77(T > C) mutation is the most common δ-globin gene mutation in China (Liu et al., 2013; Chen M. et al., 2020) and may cause δ^0^- or δ^+^-thalassemia. In our case, the -77(T > C) mutation was combined with the CD17(A > T) mutation, resulting in normal levels of Hb A2 (2.7%). This indicates that the diagnosis of β-thalassemia could be hindered when it is combined with δ-thalassemia. Moreover, we made the first discovery in the Chinese population of the novel δ-globin gene mutation CD44(TCC > TGC) (HBD:c.134C > G). The mutation at codon 44, which results in Ser→Cys acid substitution, is believed to cause δ^+^-thalassemia.

We conducted an extensive analysis of the hemoglobinopathy spectrum for the subjects in our study and identified three types of hemoglobinopathies that were induced by an α-globin gene mutation. Among them, Hb Q-Thailand (α74: Asp→His) has previously been reported in Chinese and other Southeast Asian populations. It is invariably linked to a leftward single α-globin gene deletion (–α^4.2^) and causes Hb Q-H disease when associated with α-thalassemia (mainly –^*SEA*^) (Hu et al., 2011; Zeng et al., 1992). Moreover, our study presented the first cases of Hb Owari (α121:Val→Met) in Fujian province, which was first identified in the Japanese population and exhibits normal functional properties (Harano et al., 1986). Additionally, we detected 37 cases of Hb New York, 12 cases of Hb J-Bangkok, and one case each of Hb Miyashiro and Hb G-Coushatta, all of which are induced by the β-globin gene mutation. We detected the first cases in the Quanzhou region of Hb New York, Hb J-Bangkok, and Hb G-Coushatta in this study. Previous studies indicated that co-inheritance of Hb New York with three α-globin gene deletions could lead to a severe Hb H disease (Chan et al., 1987). In this study, we identified a male compound of Hb J-Bangkok and CD41-42(-TCTT). He exhibited normal hemoglobin values (137 × 10^12^ g/L), low levels of MCV (56 fl) and MCH (18.3 pg), and increased Hb A2 (6.0%). Our results were consistent with those of a previous study, which suggested that the coexistence of Hb J-Bangkok and β-thalassemia may not aggravate the phenotype (Zhao et al., 2013). Moreover, our study first identified the rare Hb Miyashiro mutation in the Chinese population. There is a GTT > GGT mutation at codon 23 in the β-globin gene, which can be detected by polyacrylamide gel isoelectric focusing (IEF) or reversed-phase high-performance liquid chromatography (HPLC) (Nakatsuji et al., 1981; Ohba et al., 1984). Therefore, we believe that it is essential to identify the hemoglobin variants in a population with a high prevalence of thalassemia in a routine setting.

A diverse range of rare thalassemia mutations was identified in this study, which is consistent with the location and the increasing population in Quanzhou prefecture. Notably, with thalassemia detection kits, we identified some rare thalassemia mutations that were more pervasive than the so-called common thalassemia mutations, such as CD31(-C), –32(C > A), and –30(T > C), which have never been detected in this region before. Nowadays, next-generation sequencing is being increasingly used to improve thalassemia detection (He et al., 2017; Zhao et al., 2020). Thus, DNA sequencing technology combined with gap-PCR can detect all known and unknown mutations in the α- and β-globin gene in a cost-effective way, which will greatly reduce missed diagnoses.

## Conclusion

In this study, we conducted a comprehensive large-scale study on the thalassemia mutation in Quanzhou prefecture, which provides valuable data for the prevention and control of thalassemia. Our study is the first to reveal the spectrum of rare thalassemia mutations and hemoglobinopathies in the Quanzhou region. From this study, a diversity of rare thalassemia mutations and hemoglobinopathies was identified. This research, combined with the use of DNA sequencing and gap-PCR technology, shows great value in the investigation of rare and novel thalassemia gene mutations.

## Data Availability Statement

The raw data supporting the conclusions of this article will be made available by the authors, without undue reservation.

## Ethics Statement

The studies involving human participants were reviewed and approved by the Ethics Committee of The Women’s and Children’s Hospital of Quanzhou (2020No.8). The patients/participants provided their written informed consent to participate in this study.

## Author Contributions

JZ designed the study and wrote the article. NZ and JZ performed conventional thalassemia analysis. YZ performed specific gap-PCR amplification and DNA sequencing. NZ and HZ analyzed the data. YX, YJ, DC, and YW revised and polished the manuscript. All authors have approved the final article.

## Conflict of Interest

YZ was employed by the company Yaneng BIOscience (Shenzhen) Co., Ltd. The remaining authors declare that the research was conducted in the absence of any commercial or financial relationships that could be construed as a potential conflict of interest.

## Publisher’s Note

All claims expressed in this article are solely those of the authors and do not necessarily represent those of their affiliated organizations, or those of the publisher, the editors and the reviewers. Any product that may be evaluated in this article, or claim that may be made by its manufacturer, is not guaranteed or endorsed by the publisher.
